# Image Generation from Text Using StackGAN with Improved Conditional Consistency Regularization

**DOI:** 10.3390/s23010249

**Published:** 2022-12-26

**Authors:** Rihito Tominaga, Masataka Seo

**Affiliations:** Osaka Institute of Technology, Graduate School of Robotics and Design Engineering, 1-45 Chayamachi, Kita-ku, Osaka 530-0013, Japan

**Keywords:** deep learning, multimodal learning, natural language processing, image generation

## Abstract

Image generation from natural language has become a very promising area of research on multimodal learning in recent years. In recent years, the performance of this theme has improved rapidly, and the release of powerful tools has caused a great response in various places. The Stacked Generative Adversarial Networks (StackGAN) model is a representative method to generate images from text descriptions. Although it can generate high-resolution images, it involves several limitations; some of the images generated are typically unintelligible, and mode collapse may occur. Therefore, in this study, we aim to solve these two problems to generate images that follow a given text description more closely. First, we incorporate a new consistency regularization technique for conditional generation tasks into StackGAN, called Improved Consistency Regularization or ICR. The ICR technique learns the meaning of data by matching the semantic information of input data before and after data augmentation, and can also stabilize learning in adversarial networks. In this research, this method mainly suppresses mode collapse by expanding the variation of generated images. However, this method may lead to excessive variations in the generated images, which may result in images that do not match the meaning of the input text or that are ambiguous. Therefore, we further propose a new regularization method called ICCR as a modification of ICR, which is designed to perform conditional generation tasks and eliminate the negative impacts of the generator. This method realized the generation of various images along the input text. The proposed StackGAN with ICCR performed 16% better than StackGAN and 4% better than StackGAN with ICR and AttnGAN on the Inception Score using the CUB dataset. AttnGAN, similar to StackGAN, is a GAN-based text-to-image model that incorporates the attention mechanism, which has achieved great results in recent years. It is very important that our proposed model, which incorporates ICCR into a simple model, obtained better results than AttnGAN. In addition, StackGAN with ICCR was effective in eliminating mode collapse. The probability of mode collapse in the original StackGAN was 20%, while in StackGAN with ICCR the probability was 0%. In the questionnaire survey, our proposed method was rated 18% higher than StackGAN with ICR. This indicates that ICCR is more effective for conditional tasks than ICR.

## 1. Introduction

Since the advent of artificial intelligence technology, making computers perform creative tasks has been a major goal for engineers. With the remarkable development of artificial intelligence technology in recent years, it is gradually being realized. Among them, image generation is attracting a great deal of attention. Image generation technology based on artificial intelligence has made dramatic progress with Variational Auto-Encoder (VAE) [1] proposed by Kingma et al. in 2013 and Generative Adversarial Networks (GAN) [2] proposed by Ian et al. in 2014. One application of these methods is image generation based on text data. Methods to automatically generate images according to descriptions written in natural language have a wide variety of possible applications, such as art production and image editing. Driven by some notable advances, image generation from natural language has also become one of the most active areas of research on multimodal learning in recent years. On the other hand, DALL-E and DALL-E 2 [3] released by OpenAI in 2021 and Stable Diffusion [4] released by CompVis in 2022 had very high performance and were used by users all over the world, but caused various social problems.

Most existing image generation methods are based on GAN models. For example, the Stacked Generative Adversarial Networks (StackGAN) [5] architecture proposed by Han et al. divides the image generation process into two stages to generate high-resolution images. However, the task of generating high-resolution images from text descriptions is very difficult due to the complexity of learning. This learning difficulty is often due to a large number of variations in correspondence between natural language and images. There are innumerable natural language expressions that express one image, and there are also innumerable images corresponding to one natural language expression. Although StackGAN has successfully generated high-resolution images of 256 × 256 pixels, unstable learning has caused problems such as unintelligible images and mode collapse. This problem becomes more pronounced as the resolution of the generated image increases in the conditional generation task.

Our prior research has shown that these problems can be mitigated by incorporating Improved Consistency Regularization (ICR) [6], a learning stabilization method, into StackGAN [7]. Our proposed method uses ICR to learn corresponding to various representations of natural language and images, and achieves a certain degree of accuracy improvement. However, ICR does not inherently support conditional generation tasks; ICR has a structure to increase the variation of generated images, but this leads the user to focus on increasing variation while ignoring conditions. To prevent this, a structure for conditional generation is needed. We, therefore, propose a new regularization method, Improved Conditional Consistency Regularization (ICCR), for conditional generation tasks. It prevents mode collapse and condition-neglected generation by constraining latent variables in the generated image to a meaning-preserving range.

## 2. Related Work

The field of data generation tasks has made great progress since GAN was first published. In the field of image generation from text, the model proposed by Reed et al. [8] was the first to successfully generate high-resolution images. Existing text-to-image GANs convert the entire sentence into a single vector and use it. AttnGAN [9] generates a region that is most closely related to a specific word among finely segmented regions in an image. As a result, AttnGAN achieved better accuracy than existing methods. Recently, the Diffusion Model [3,4,10,11] has been attracting attention. The Latent Diffusion Model [12] has improved the stability of learning and succeeded in generating higher-resolution images compared to adversarial learning. These methods sample the latent variables from the learned distributions, and the similarities of the algorithms to VAE can be seen.

On the other hand, GAN-based models generally produce clearer images than VAE-based models. Therefore, in this research, we introduced our proposed method to a GAN-based text-to-image model and conducted an experiment.

### 2.1. Generative Adversarial Networks

GAN architectures use two separate neural network models to generate data via an adversarial learning process, which are referred to as generator and discriminator models. The generator takes random noise z as input and generates data, and the discriminator then classifies the input data as being real or fake, that is, synthetic data generated by the generator. The discriminator is designed to learn instances of the source data in contrast to the output of the generator, whereas the generator learns to generate data that can fool the discriminator. These two models solve the optimization problem given below in Equation (1).

(1) $$\begin{array}{c} \underset{G}{\min}\;\underset{D}{\max}V\left(G,D\right)=E_{x\sim p_{data}\left(x\right)}\log D\left(x\right)+E_{z\sim p\left(z\right)}\log(1-D\left(G\left(z\right)\right)). \end{array}$$

The random noise z input to the generator is sampled from an arbitrary distribution $p\left(z\right)$ in the latent space. In contrast, the input to the discriminator is either x sampled from the observed data distribution $p_{data}\left(x\right)$ or the generated data $G\left(z\right)$.

### 2.2. Stacked Generative Adversarial Networks

**Figure 1:**
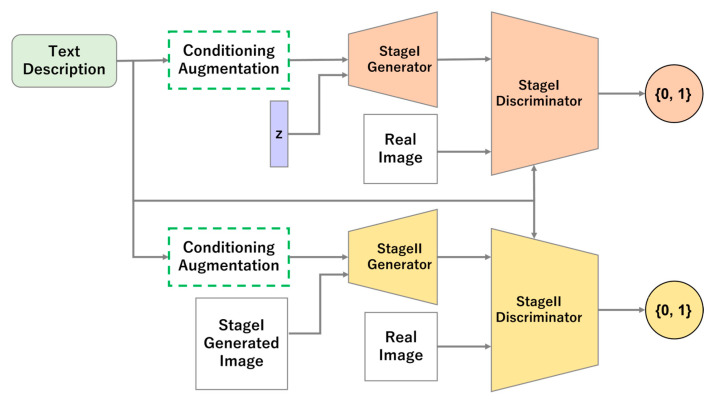
Overview of the Stacked Generative Adversarial Networks model.

The StackGAN model takes a text description as input and generates images that capture its features through a two-step process. Stage I generates low-resolution images that capture features such as rough shapes and colors. In Stage II, the images output by the trained Stage I model are input to the generator, which then generates a high-resolution image with the features not represented in Stage I. High-resolution image generation using this two-step process follows the approach of pgGAN [13]. An overview of StackGAN is shown in Figure 1.

Stage II includes three routes from the input to the discriminator. An input image may comprise observed data matching the input text; alternatively, the input image may be observed data that does not match the input text. Finally, the input image may also be synthetic, having been generated by the generator in Stage I.

#### 2.2.1. Conditioning Augmentation

The latent variable vector $\varphi_{t}$ of text input is often high-dimensional (>100), which hinders stable learning. Hence, our proposed method assumes that the distribution of the latent space is Gaussian and $\hat{c}$ is sampled randomly from the Gaussian distribution $N\left(\mu_{0}\left(\varphi_{t}\right),\sigma \left(\varphi_{t}\right)\right)$ and used as an input to the generator. When the latent space becomes high-dimensional, the mapping to the latent variables becomes correspondingly sparse, and acquiring features becomes difficult. However, limiting the distribution of the latent space to a Gaussian distribution can solve this problem by increasing the density of the mapping of latent variables.

#### 2.2.2. Stage I

Stage I generates low-resolution images that capture the broad features of the text description. The input to the generator comprises $\hat{c}$ sampled from the latent variable space of the conditioning augmentation and z sampled from an arbitrary distribution $p\left(z\right)$. The discriminator does not consider whether the input image is real at this stage; rather, it classifies images according to whether they match the text description. Hence, there are three input patterns as noted above, including authentic images that match the input text description, authentic images that do not match, and generated images. The discriminator’s loss function is shown in Equation (2), and that of the generator is shown in Equation (3).

(2) $$\begin{array}{c} L_{D_{0}}=-E_{\left(I_{0},t\right)\sim p_{data}}\left[\log D_{0}\left(I_{0},\varphi_{t}\right)\right]-E_{z\sim p\left(z\right),t\sim p_{data}}\left[\log\left(1-D_{0}\left(G_{0}\left(z,\hat{c}\right),\varphi_{t}\right)\right)\right], \end{array}$$

(3) $$\begin{array}{cc} L_{G_{0}}= & E_{z\sim p\left(z\right),t\sim p_{data}}\left[\log\left(1-D_{0}\left(G_{0}\left(z,\hat{c}\right),\varphi_{t}\right)\right)\right]\\ & +\lambda_{0}D_{KL}\left(N\left(\mu_{0}\left(\varphi_{t}\right),\sigma \left(\varphi_{t}\right)\right)\|N\left(0,1\right)\right), \end{array}$$

where the real image $I_{0}$ and the text description t are obtained from the observed data distribution $p_{data}$, z is a random sample from the distribution $p\left(z\right)$, and $\lambda_{0}$ is a hyperparameter.

#### 2.2.3. Stage II

Stage II generates a high-resolution image to correct the image generated in Stage I to render detailed features. In Stage II, the inputs to the generator comprise $\hat{c}$ sampled from Stage I’s conditioning augmentation and $s_{0}$ from the images generated by Stage I. Therefore, the loss functions of the discriminator and generator in Stage II are as shown in Equations (4) and (5), respectively.

(4) $$\begin{array}{c} L_{D}=-E_{\left(I,t\right)\sim p_{data}}\left[\log D_{0}\left(I,\varphi_{t}\right)\right]-E_{s_{0}\sim p_{G_{0}},t\sim p_{data}}\left[\log\left(1-D\left(G\left(s_{0},\hat{c}\right),\varphi_{t}\right)\right)\right], \end{array}$$

(5) $$\begin{array}{ll} L_{G}= & E_{s_{0}\sim p_{G_{0}},t\sim p_{data}}\left[\log\left(1-D\left(G\left(s_{0},\hat{c}\right),\varphi_{t}\right)\right)\right]\\ & +\lambda D_{KL}\left(N\left(\mu_{0}\left(\varphi_{t}\right),\sigma \left(\varphi_{t}\right)\right)\|N\left(0,1\right)\right), \end{array}$$

### 2.3. Improved Consistency Regularization

ICR is based on consistency regularization (CR). CR methods are designed to stabilize the learning processes of generative adversarial models. CR adds the following consistency regularization expression to the loss function of the discriminator model.

(6) $$\begin{array}{c} L={\left\|D\left(x\right)-D\left(T\left(x\right)\right)\right\|}^{2}, \end{array}$$

where $T\left(x\right)$ represents the data augmentation (DA) of the observed data x. Because the DA of the observed data does not alter its original meaning, the presence or absence of DA should not significantly affect the mapping by the discriminator to the latent space. Therefore, by providing a loss function that reduces the difference in the discriminator’s latent variables owing to the presence or absence of DA, the latent space learns the semantic information of the input data to improve the performance of the discriminator. However, CR involves some challenges, which ICR was proposed to solve in addition to providing improved performance. ICR combines balanced consistency regularization (bCR) and latent consistency regularization (zCR).

#### 2.3.1. Balanced Consistency Regularization

**Figure 2:**
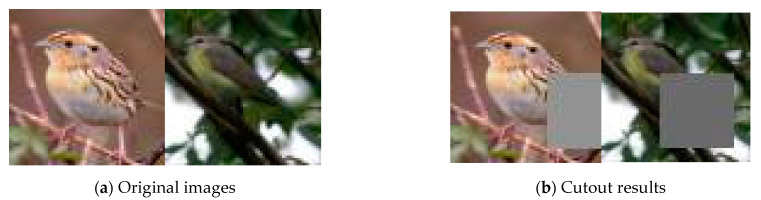
Cutout processing.

Incorporating CR improves the performance of GAN models, and bCR was proposed to address some limitations of this approach. An example of DA is shown in Figure 2, in which a cutout masks part of the image. In this case, the generator also learns to process masks as a feature of the observed data, and the generated images may thus include apparent cutouts.

**Figure 3:**
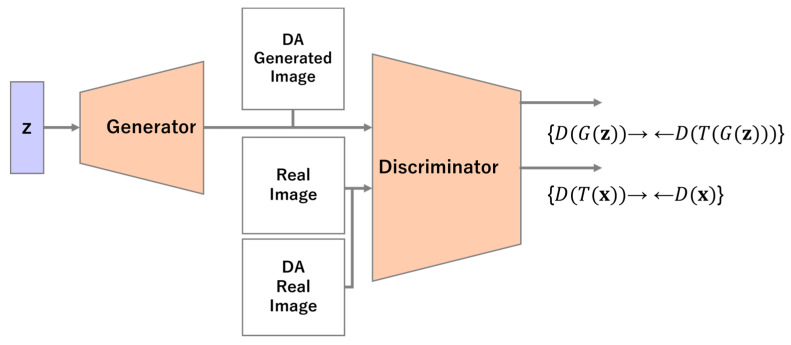
Overview of balanced consistency regularization.

To solve this problem, bCR performs DA on the output data of the generator. An overview of bCR is shown in Figure 3.

The loss function of the discriminator is supplemented with expressions for the observed (7) and generated (8) data as given below.

(7) $$\begin{array}{c} L_{real}={\left\|D\left(x_{real}\right)-D\left(T\left(x_{real}\right)\right)\right\|}^{2}, \end{array}$$

(8) $$\begin{array}{c} L_{fake}={\left\|D\left(x_{fake}\right)-D\left(T\left(x_{fake}\right)\right)\right\|}^{2}, \end{array}$$

Therefore, the loss function of the discriminator is as follows.

(9) $$\begin{array}{c} L_{D}^{bcr}=L_{D}+\lambda_{real}L_{real}+\lambda_{fake}L_{fake}, \end{array}$$

where $\lambda_{real}$ and $\lambda_{fake}$ are hyperparameters.

#### 2.3.2. Latent Consistency Regularization

**Figure 4:**
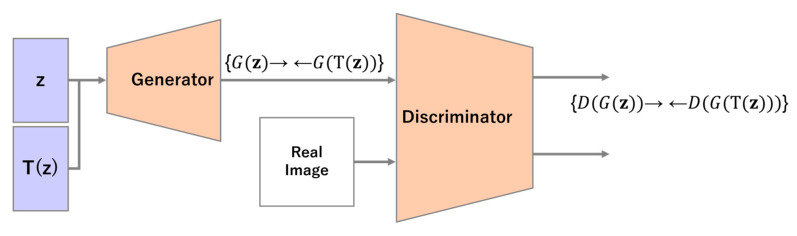
Overview of latent consistency regularization.

In bCR, DA is performed on the input of the discriminator, whereas in zCR, DA is performed on noise z, which is the input of the generator. An overview of zCR is shown in Figure 4.

To improve the performance of the discriminator by enforcing consistency in the noise space, the hypothesis that the discriminator output should be consistent is incorporated when DA is applied to noise z. The generator is given two input patterns, including z with and without the DA. The following expression is added to the loss function to bring the two outputs of the generator closer when they are input to the discriminator.

(10) $$\begin{array}{c} L_{dis}={\left\|D\left(G\left(z\right)\right)-D\left(T\left(G\left(z\right)\right)\right)\right\|}^{2}, \end{array}$$

Therefore, the loss function of the discriminator is given as follows.

(11) $$\begin{array}{c} L_{D}^{zcr}=L_{D}+\lambda_{dis}L_{dis}, \end{array}$$

However, this approach is prone to mode collapse, where the generator produces the same image, regardless of z. The following expression is added to the loss function of the generator to diversify its output.

(12) $$\begin{array}{c} L_{gen}=-{\left\|G\left(z\right)-G\left(T\left(z\right)\right)\right\|}^{2}, \end{array}$$

Therefore, the loss function of the generator is given as

(13) $$\begin{array}{c} L_{G}^{zcr}=L_{G}+\lambda_{gen}L_{gen}, \end{array}$$

where $\lambda_{dis}$ and $\lambda_{gen}$ are hyperparameters.

## 3. StackGAN with ICCR

ICR maximizes the L2 norm among the generated data to increase the amount of variation they contain. This may cause a problem in that ICR maximizes the L2 norm even when the same conditions are given for the generation, resulting in the generation of images that are far from the given conditions. Therefore, we propose StackGAN with ICCR as a new consistency regularization method for conditional generative models.

### 3.1. Stage I

**Figure 5:**
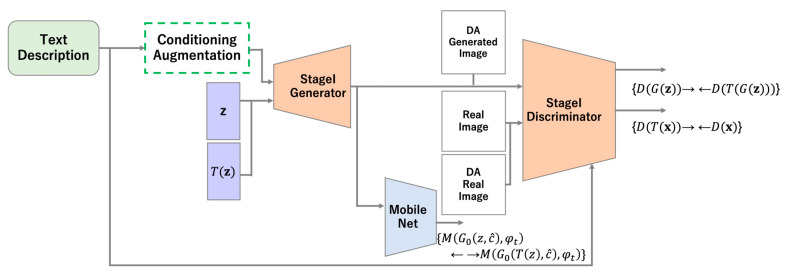
Diagram of Stage I of StackGAN with ICCR.

The training process for Stage I of StackGAN with ICCR is performed using a MobileNet v1 model pre-trained on the ImageNet dataset to obtain the latent variables corresponding to the generated images. In ordinary ICR, the latent variable space of the generator is used; however, by using a pre-trained MobileNet model, latent variables that capture features can be used even in the early stages of training to improve the stability of the process. The fact that the evaluation criteria do not change as learning progresses also contributes to improved stability. A diagram of the proposed model is shown in Figure 5; the loss function of the discriminator is given in Equation (14), and that of the generator is provided in Equation (15).

(14) $$\begin{array}{ll} L_{D_{0}}^{ICCR}= & -E_{\left(I_{0},t\right)\sim p_{data}}\left[\log D_{0}(I_{0},\varphi_{t})\right]\\ & -E_{z\sim p\left(z\right),t\sim p_{data}}\left[\log(1-D_{0}(G_{0}\left(z,\hat{c}\right),\varphi_{t}))\right]\\ & +\alpha {\left\|D_{0}\left(I_{0}\right)-D_{0}\left(T\left(I_{0}\right)\right)\right\|}^{2}\\ & +\alpha {\left\|D_{0}\left(G_{0}\left(z,\hat{c}\right),\varphi_{t}\right)-D_{0}\left(T\left(G_{0}\left(z,\hat{c}\right),\varphi_{t}\right)\right)\right\|}^{2}\\ & +\beta {\left\|D_{0}\left(T(G_{0}\left(z,\hat{c}\right)\right),\varphi_{t})-D_{0}\left(G_{0}\left(T\left(z\right),\hat{c}\right),\varphi_{t}\right))\right\|}^{2}, \end{array}$$

(15) $$\begin{array}{ll} L_{G_{0}}^{ICCR}= & E_{z\sim p\left(z\right),t\sim p_{data}}\left[\log(1-D_{0}(G_{0}\left(z,\hat{c}\right),\varphi_{t}))\right]\\ & +\lambda D_{KL}(N\left(\mu_{0}\left(\varphi_{t}\right),\sigma \left(\varphi_{t}\right)\right)\|N\left(0,1\right))\\ & -\gamma {\left\|M\left(G_{0}\left(z,\hat{c}\right),\varphi_{t}\right)-M\left(G_{0}\left(T\left(z\right),\hat{c}\right),\varphi_{t}\right)\right\|}^{2}, \end{array}$$

### 3.2. Stage II

Stage II generates a high-resolution image that modifies the output generated in Stage I. Therefore, the input of the generator is changed from noise z to $s_{0}$ of the image generated in Stage 1. The third term in Equation (15) can be modified as shown in Equation (16) to prevent the generation of images that do not conform to the conditions or that exhibit mode collapse.

(16) $$\begin{array}{ll} L_{G_{1}}^{ICCR}= & E_{z\sim p\left(z\right),t\sim p_{data}}\left[\log(1-D_{1}(G_{1}\left(z,\hat{c}\right),\varphi_{t}))\right]\\ & +\lambda D_{KL}(N\left(\mu_{0}\left(\varphi_{t}\right),\sigma \left(\varphi_{t}\right)\right)\|N\left(0,1\right))\\ & +\gamma \left({\left\|M\left(s_{0}\right)-M\left(T\left(s_{0}\right)\right)\right\|}^{2}-{\left\|M\left(G_{1}\left(s_{0},\hat{c}\right),\varphi_{t}\right)-M\left(T\left(G_{1}\left(s_{0},\hat{c}\right),\varphi_{t}\right)\right)\right\|}^{2}\right), \end{array}$$

**Figure 6:**
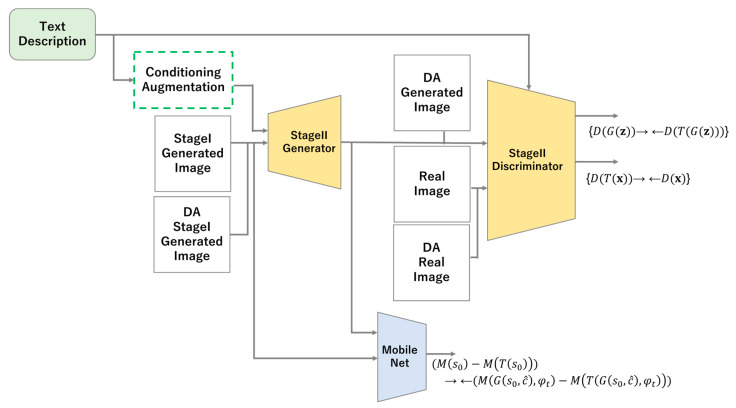
Model diagram of Stage II in StackGAN with ICCR.

This forces changes in latent variables due to DA to be retained in the generator’s output image, which prevents the generation of images that deviate significantly from the conditions or that exhibit mode collapse. A diagram of the Stage II model of StackGAN with ICCR is shown in Figure 6.

## 4. Experiments

This section describes experiments conducted to evaluate the performance of the proposed approach.

### 4.1. Experimental Setup

In this section, we describe the dataset used in the experiments as well as the experimental setup, including the hyperparameters.

#### 4.1.1. Data Set

**Figure 7:**
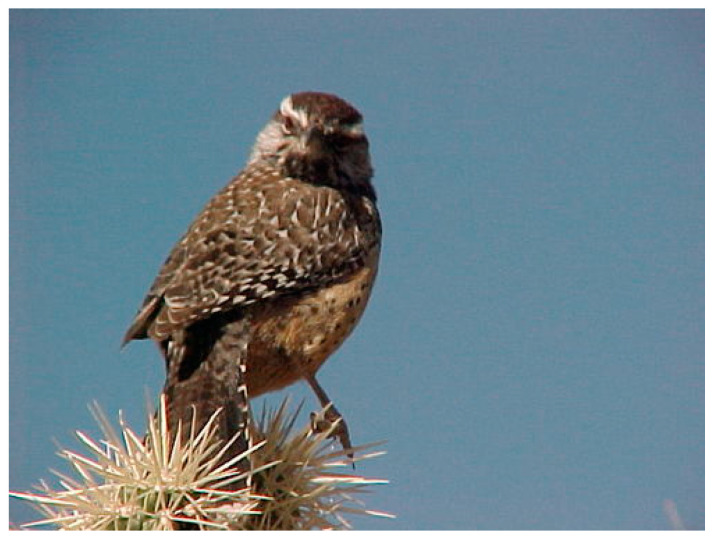
Example image from Caltech-UCSD Birds.

The Caltech-UCSD Birds [14] dataset was used in the experiment. The CUB contains a total of 11,788 images of birds of 200 different species. Each image includes a set of text describing the color, pattern, and shape of each specific area. For example, the image in Figure 7 shows a cactus wren, and the text describing this image is presented below.

- This bird has a dark brown crown, a white superciliary, and a spotted back with spotted tail feathers.

#### 4.1.2. Network Setup

600 epochs were used to train the StackGAN model for both stages I and II, and 1000 epochs were used for Stage I of StackGAN with ICR for comparison and 600 for Stage II. The number of epochs was determined by observing the values of the loss and the number of epochs until convergence for each model. The StackGAN hyperparameters, $\lambda_{0}$ and λ in Equations (3) and (4), were set to 1. This is in accordance with the values proposed in [5]. The hyperparameters of StackGAN with ICR, $\lambda_{real}$, $\lambda_{fake}$, $\lambda_{dis}$, and $\lambda_{gen}$ in Equations (9), (11), and (13), were set to $\lambda_{real}=0.1$, $\lambda_{fake}=0.1$, $\lambda_{dis}=0.5,$ and $\lambda_{gen}=0.001$, respectively. The hyperparameters in Equations (14), (15), and (16) of StackGAN with ICCR were assigned values of λ=1, α=0.1, β=0.5, γ=0.001, respectively. In this research, these hyperparameters were determined experimentally. Experiments were performed with different parameters, and it is known that they are not so sensitive. The size of the generated image was 64 × 64 pixels for Stage I and 256 × 256 pixels for Stage II.

### 4.2. Evaluation Metrics

Evaluating the performance of generative models is difficult. We used a numerical evaluation method called “Inception Score” (I) [15] to evaluate the model quantitatively, as given below.

(17) $$\begin{array}{c} I=\exp\;(E_{x}D_{KL}\left(p\left(y|x\right)\left|\right|p\left(y\right))\right), \end{array}$$

where x is a single generated sample and y is the label predicted by the Inception-v3 model [16]. The reasoning behind this measure is that a good model should produce diverse but meaningful images. Therefore, the KL divergence between the marginal p(y) and the conditional $p\left(y|x\right)$ distributions should be large. We also evaluated this measure on 3000 randomly selected samples for each model.

### 4.3. Experimental Results

In this section, we describe the experimental results. We used the generated images, the incidence of mode collapse, Inception Score, and the results of a questionnaire as measures to compare the accuracy of the generated content. We compared StackGAN, StackGAN with ICR, AttnGAN, and StackGAN with ICCR models. 

#### 4.3.1. Comparison of Generated Images

**Figure 8:**
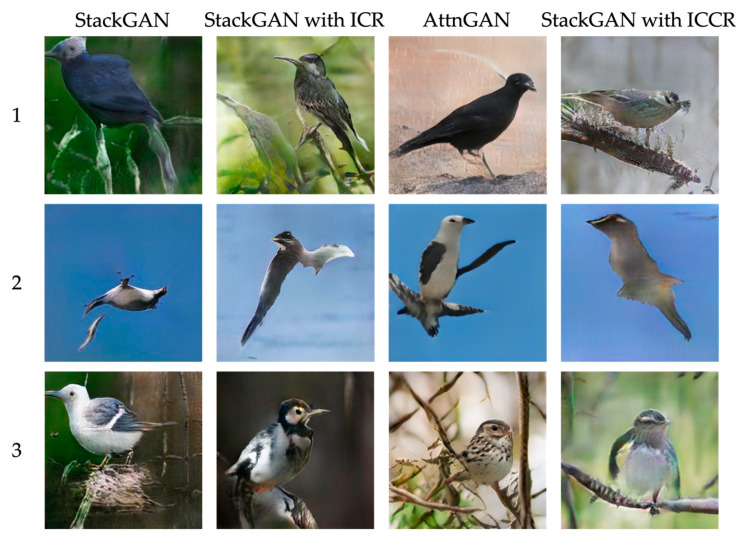
Images generated by each model.

The images generated by the four models are shown in Figure 8. The generated images for StackGAN, with ICR, and with ICCR were obtained from Stage II. The following four texts were used as inputs.

1. This black bird has no crest, a medium-pointed bill, and a short tail.
2. This is a white bird with black wings and a small beak.
3. This small bird has a white belly and breast, and is mostly speckled otherwise.

The results for the text labeled 1 show that the AttnGAN model was able to generate images that captured the features of the input data best. StackGAN also succeeded in generating images that captured color features, although the bird shape was slightly distorted. For text 2, all models failed to generate an image. For text 3, StackGAN and StackGAN with ICCR were able to capture the “white belly and breast” features. Only AttnGAN was able to represent “mostly speckled otherwise”. 

#### 4.3.2. Incidence of Mode Collapse

**Figure 9:**
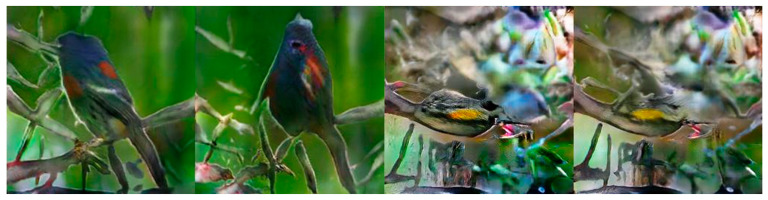
Example of mode collapse occurring in StackGAN.

We compared StackGAN and StackGAN with ICCR in terms of the incidence of mode collapse. Four types of input noise z were used for a single input text. In total, images were generated for 20 texts. In this experiment, we observed mode collapse in the output for four texts with StackGAN, as shown in Figure 9, and no instances of mode collapse were observed with StackGAN with ICCR.

#### 4.3.3. Comparison of Inception Score

The inception scores of each model are listed in Table 1. StackGAN, StackGAN with ICR, and StackGAN with ICCR were trained in three trials and the mean and standard deviation of the Inception Score were recorded. For AttnGAN, we downloaded the trained model from GitHub. Therefore, the mean and standard deviation were obtained from three trials of the same trained model with different input noise.

As shown in Table 1, StackGAN with ICCR had the highest Inception Score value. Specifically, it performed approximately 16% better than StackGAN, 4% better than StackGAN with ICR, and 4% better than AttnGAN.

#### 4.3.4. Comparison by Questionnaire Survey

A survey was conducted with 21 people using images generated by StackGAN with ICR and StackGAN with ICCR. Twenty texts were entered into each model, and the survey asked which of the output images were clearer. In this case, we did not reveal which output image was produced by which model. The results showed that 41% of the respondents chose StackGAN with ICR as clearer and 59% chose StackGAN with ICCR. 

## 5. Discussion

### 5.1. Conclusions

As a result of comparing the images generated by each model, the results of the proposed model in this research were not good in all trials. By comparing a large number of generated images, we found that our proposed model is statistically superior. The images generated by StackGAN showed mode collapse in 4 out of 20 texts, whereas those produced by StackGAN with ICCR showed no mode collapse in 20 texts. This indicates that introducing ICCR may be expected to eliminate mode collapse. StackGAN with ICCR also had the highest Inception Score value among the four models. The standard deviation was the smallest, indicating that the training process was stable. StackGAN with ICR, the model with the second-highest Inception Score, was used in the survey for comparison, and StackGAN with ICCR obtained higher values in terms of human perception. This shows that the ICCR is more effective than ICR in the conditional generation model.

### 5.2. Recommendation

In the future, we plan to establish a stable learning method for GAN models, which is generally considered challenging, and to build a system that can handle text input describing more complex scenes. The text contained in the Caltech-UCSD Birds we used was relatively simple. Thus, we also want to achieve more complex image generation from natural language.

## Figures and Tables

**Table 1:** Inception Score of each model.

Model	Inception Score
StackGAN	4.75 ± 0.16
StackGAN with ICR	5.30 ± 0.15
StackGAN with ICCR	5.51 ± 0.05
AttnGAN	5.32 ± 0.11

## Data Availability

Not applicable.
